# The Bifactor Structure of the Emotion Expression Scale for Children in a Sample of School-Aged Portuguese Children

**DOI:** 10.1177/10731911221082038

**Published:** 2022-03-10

**Authors:** Brígida Caiado, Maria Cristina Canavarro, Helena Moreira

**Affiliations:** 1 University of Coimbra, Portugal

**Keywords:** emotion expression, bifactor model, factor structure, children

## Abstract

The Emotion Expression Scale for Children (EESC) is a 16-item self-report questionnaire assessing children’s difficulties in emotion expression (i.e., poor emotion awareness and reluctance to express emotions). Considering the inconsistent findings regarding its factorial structure and dimensionality, this study aims to explore the factor structure and psychometric properties of the EESC in a sample of 286 Portuguese children (8–12 years). Three competing models were analyzed through confirmatory factor analysis (correlated two-factor model, one-factor model, and bifactor model). The bifactor model provided a better fit than the competing models, and the results suggested a strong general factor of “difficulties in emotion expression.” The validity of the EESC was also indicated by its positive correlations with variables assessing child anxiety, depression, and behavioral avoidance and its negative correlation with mindfulness skills. The EESC is a valid measure of children’s difficulties in emotion expression and the use of its total score is recommended.

Emotion regulation is a “multi-componential” process (Gross, 2002, p. 282) that includes the ability to understand and integrate emotional information from the social environment and to evaluate and manage one’s own emotional reactions to accomplish one’s goals (Penza-Clyve & Zeman, 2002; Thompson, 1994; Zeman et al., 2006). Therefore, emotion regulation includes “intrinsic and extrinsic processes” (Thompson, 1994, p. 28), namely, emotion awareness and emotion expression, respectively (Gross, 1998, 1999, 2002; Penza-Clyve & Zeman, 2002), which are skills of emotional competence (Saarni, 1999).

Emotion awareness is a cognitive and attentional process that enables the individual to identify, label, perceive, differentiate, and monitor one’s emotional experiences (Boden & Thompson, 2015; Lane et al., 1998; Rieffe et al., 2008). It also includes an attitudinal aspect responsible for evaluating one’s and other’s emotions (e.g., as positive or negative, private or not; Rieffe & De Rooij, 2012; Rieffe et al., 2008). Emotion expression refers to the ability to convey emotional experience into emotional reactions (verbal or nonverbal) moderating its intensiveness and direction in a balanced way to accomplish one’s goals (Gross, 1998, 1999; Mayer & Salovey, 1997; Penza-Clyve & Zeman, 2002). Neural correlates of emotion awareness are distinct from emotion expression (Lane et al., 1998) as emotion awareness is an intrinsic process and does not necessarily involve an outward display, whereas emotional expression is an extrinsic process that is responsible for emotional reactions and the expression or repression of emotions (Croyle & Waltz, 2002; Penza-Clyve & Zeman, 2002).

More specifically, emotion awareness and emotion expression can be conceptualized as different stages in the emotion regulation process. Emotion awareness could represent an early important stage of the emotion regulation process, as prior to initiating emotion regulation strategies and expressing emotions, one may first need to note the presence of an emotional state that needs to be regulated (Gohm & Clore, 2002; Gross, 1998, 1999). Moreover, as emotion awareness is related to the development of beliefs about emotions, this process could lead to the development of beliefs about whether emotions should be expressed or repressed (Ford & Gross, 2019; Penza-Clyve & Zeman, 2002; Rieffe & De Rooij, 2012; Rieffe et al., 2008). Hence, this concept is closely related to emotional expression and is an important prerequisite for the motivation to express or repress emotion and the consequent activation of a certain behavioral, cognitive, and emotional response (Barrett et al., 2001; Penza-Clyve & Zeman, 2002; Saarni, 1999). Therefore, emotion expression—as a general concept—involves both the recognition and the awareness of internal experiences and the modulation of emotional responses (Gross & Thompson, 2007; Long et al., 2013). In line with this view, Penza-Clyve and Zeman (2002) suggested that poor emotion awareness and reluctance to express emotions would be part of a broader concept of emotion expression.

## The Role of Emotion Expression in Children’s Mental Health

The emotion socialization process occurs in the context of the parent–child relationship. Namely, through parental modeling of emotions and the way that parents teach about emotions, children learn about the expression, function, and consequences of emotions (Eisenberg et al., 1998a, 1998b). Thus, during the emotional socialization process, children transition from co-regulation processes (more dependent on parents) toward greater self-regulation, acquiring initial strategies for modulating and expressing emotions. At the age of 9 to 12 years old, children show an increased awareness of the complexity of emotional experiences, expressing their emotions according to their environment, considering the impact that this expression could have on others, and beginning to understand that the same experience can elicit both positive and negative emotional reactions. Simultaneously, as a result of social interaction and cognitive development, children develop the ability to reflect about emotions more abstractly, to conceptualize and verbalize ideas about them, and acquire new cultural/social rules of conduct, which are important for their understanding of emotions and for their motivation or reluctance to express emotions (Cole et al., 1994; Henderson et al., 2017).

The ability to successfully regulate and express emotions is central to psychological health, social development, and academic achievement in children (e.g., Gross, 2002; Spinrad et al., 2006; Trentacosta & Izard, 2007), and it is a protective factor against the negative impact of negative life events (Casey, 1996; Shields & Cicchetti, 2001; Zeman et al., 2002). On the contrary, poor emotion regulation skills have consistently been associated with a wide range of internalizing and externalizing symptoms in children and adolescents (Silk et al., 2003; Suveg & Zeman, 2004; Walcott & Landau, 2004) and are considered a potential transdiagnostic mechanism of psychopathology (Harvey et al., 2015; Kring & Sloan, 2010; Moses & Barlow, 2006).

Poor emotion awareness, a component of emotional expression (Gross & Thompson, 2007; Penza-Clyve & Zeman, 2002), has been associated, in cross-sectional and longitudinal studies, with higher levels of depression, anxiety, and somatic complaints (Kranzler et al., 2016; Penza-Clyve & Zeman, 2002; Suveg et al., 2009; Zeman et al., 2002). Emotion awareness has also been identified in empirical studies as an important process for children’s mental health, namely, for fewer symptoms of rumination and worry (Rieffe & De Rooij, 2012). Therefore, emotion awareness has been identified as a potential transdiagnostic mechanism of psychopathology (Gross & John, 2003; Harvey et al., 2015; Kranzler et al., 2016; Kring & Sloan, 2010; Moses & Barlow, 2006) and has been implicated in a range of disorders with internalizing symptoms (e.g., childhood anxiety disorders; Southam-Gerow & Kendall, 2000), acute depression (Berthoz et al., 2000), eating disorders (Sim & Zeman, 2004, 2006)), externalizing disorders (e.g., oppositional defiant and conduct disorder (Casey, 1996; Factor et al., 2016), and a higher risk of comorbid disorders (Factor et al., 2016).

On the contrary, maladaptive expression of emotions (e.g., suppression of emotional expression; reluctance to express emotions; lack of positive emotional expression) has been linked to externalizing and internalizing problems (Keltner et al., 1995; Lougheed & Hollenstein, 2012; Zeman et al., 2002). Specifically, reluctance to express emotions has been associated with higher levels of anxiety (Penza-Clyve & Zeman, 2002; Suveg & Zeman, 2004; Zeman et al., 2001), higher levels of cognitive distortions (Scott et al., 2018), social isolation, lower social skills competence, and impaired social relationships in childhood (Jacob et al., 2014; Scott et al., 2018). It has also been identified as a risk factor for adolescent depression (Betts et al., 2009; Feng et al., 2009; Larsen et al., 2013).

## The Emotion Expression Scale for Children

Penza-Clyve and Zeman (2002) developed the Emotion Expression Scale for Children (EESC), a self-report questionnaire composed of 16 items assessing children’s difficulties in emotion expression, namely, difficulties with being aware of one’s emotions (Emotion awareness subscale) and the lack of motivation or reluctance to express emotions (Expressive Reluctance subscale). The items of the EESC were based, in part, on the Toronto Alexithymia Scale for adults (Bagby et al., 1986), which is a scale that accesses impoverished ability to express emotion. Then, in a sample of 208 children aged between 9 and 12 years, the authors conducted a principal component analysis with varimax rotation, which yielded a two-factor structure: (a) Poor Awareness (8 items describing a lack of emotion awareness) and (b) Expressive Reluctance (8 items describing an unwillingness to express emotion). Both subscales presented a good internal consistency (Poor Awareness, α = .83; Expressive Reluctance, α = .81) but a poor test–retest reliability (Poor Awareness, *r* = .59; Expressive Reluctance, *r* = .56). Its construct validity was suggested by the significant and positive correlations between Poor Awareness and Expressive Reluctance factors and measures of internalizing symptoms (depression, anxiety, and somatization), sadness and anger management, and control of emotional expression in the presence of a peer.

## The Utility of EESC in the Study of Children’s Mental Health

The EESC has allowed the development of studies aimed at understanding the transdiagnostic mechanisms that underlie the socioemotional difficulties of children and, consequently, the development of effective interventions to treat children’s psychopathology and promote children’s mental health. Indeed, the EESC has been widely used in several cross-sectional and longitudinal studies (e.g., Kranzler et al., 2016; McLaughlin et al., 2011; Scott et al., 2018) with clinical (e.g., Queen & Ehrenreich-May, 2014; Trosper & Ehrenreich May, 2011) and nonclinical samples (e.g., Brockenberry, 2016; Long et al., 2013), and it has been used in studies assessing the efficacy of intervention programs (e.g., Allen et al., 2012; Hammond et al., 2009). This corroborates the EESC’s qualities and demonstrates its usefulness and contribution to scientific research on emotion expression.

Significant associations between EESC factors and measures of depressive and anxiety symptomatology have been consistently found in empirical studies, both cross-sectionally (Brockenberry, 2016; Kranzler et al., 2016; Scott et al., 2018) and longitudinally (e.g., McLaughlin et al., 2011). Brockenberry (2016) also found that both EESC factors predicted higher levels of children’s depressive symptoms. Congruently, Kranzler et al. (2016) found that low emotion awareness predicted both depressive and anxiety symptoms. Specifically, the authors found that for each unit decrease in children’s emotion awareness levels, the risk of experiencing an increase in depression and anxiety symptomatology increases by approximately twofold. Moreover, in this study, the emotion awareness factor (measured by EESC) also mediated both the cross-sectional and the longitudinal associations between anxiety and depressive symptoms, emerging as a transdiagnostic risk factor and suggesting that emotion awareness may help explain concurrent symptoms of depression and anxiety (cross-sectional association) and the progression from anxiety to depressive symptoms (longitudinal association).

In addition, both EESC factors were positively associated with higher difficulties in managing children’s anger, with a major tendency to inhibit the expression of their feelings of sadness (Christian, 2012), and the emotion awareness factor was associated with higher levels of rumination (McLaughlin et al., 2011). Higher levels of reluctance to express emotions were also correlated with higher levels of cognitive distortions (namely, social and academic cognitive distortions) and lower levels of social skill competence (Scott et al., 2018) and playfulness (i.e., child’s spirit to play; Christian, 2012).

## The EESC: One-Factor or Two-Factor Structure?

Although Penza-Clyve and Zeman (2002) found a two-factor structure of the EESC (Poor Awareness and Expressive Reluctance factors), the results of the following studies are not congruent, raising doubts about whether EESC is better represented by two subscales or by a single factor.

Namely, the EESC’s psychometric properties were recently investigated by Nitkowski et al. (2019) in a study aimed at validating the German version of the questionnaire. In a sample of 588 adolescents (aged 10–15 years), the authors conducted a confirmatory factor analysis (CFA) to examine a correlated two-factor model, a hierarchical model, and a single-factor model. The hierarchical model presented an unacceptable fit and thus was rejected. The correlated two-factor model and the single-factor model presented similar fits but were also not good enough to be retained. Thus, the authors conducted an exploratory factor analysis (EFA), which, contrary to the proposal of the original EESC study, yielded a one-factor structure composed of 14 items (Items 4 and 6 were eliminated as they did not substantially correlate with any factor). The single-factor model, composed of 14 items, was analyzed by CFA. Item 15 was deleted as it was considered redundant with Item 9. The final single-factor model composed of 13 items presented a satisfactory fit. The factor was named “Low Emotion Awareness/Suppression” and had a Cronbach’s α value of .83.

Nitkowski et al.’s (2019) study was the only one to validate the original EESC among a different population. However, some previous studies also explored its factor structure without aiming to validate the EESC, thereby contributing to the debate around its uni- or bidimensionality. For instance, Desrosiers et al. (2015), in a study aimed to examine the associations between emotion expression and substance use in a sample mainly composed of African American and Hispanic adolescents and young adults, also found a one-factor structure of the EESC (through an EFA) and high correlations between Emotion Awareness and Expressive Reluctance subscales. In the same way, Long et al. (2013), in a study that aimed to examine emotion expression and sibling-parent emotion communication among Latino and non-Latino White siblings of children with intellectual disabilities, also found a one-factor structure of EESC for Latino children through an EFA.

Therefore, there is still no consensus on the dimensionality of the scale, that is, on whether the EESC is best represented by a single factor or by the two factors proposed by the authors of the scale. This lack of consensus is reflected in an inconsistent use of the total score or the two subscales of the instrument. Indeed, some studies used the scores of the two subscales independently (e.g., Allen et al., 2012; Brockenberry, 2016; Christian, 2012; Hammond et al., 2009; Queen & Ehrenreich-May, 2014), others used only one specific subscale (e.g., Scott et al., 2018), others used only the total score (Desrosiers et al., 2015; Long et al., 2013; Trosper & Ehrenreich May, 2011), and others used both the total score and the subscales (Choi & Lee, 2015). Thus, more research is clearly needed to clarify the factorial structure and dimensionality of the EESC and ascertain whether its two subscales or the total score should be used. This clarification may be psychometrically and theoretically relevant. First, this would standardize the use of the EESC (for clinical and research purposes). Second, this would give empirical support for the uni- or bidimensionality of the concept of Emotion Expression.

## The Current Study

Although the EESC is a relevant and widely used scale, it was validated only among American children (Penza-Clyve & Zeman, 2002) and German adolescents (Nitkowski et al., 2019). Thus, the validation of this scale to another culture seems relevant to contribute to the validity of the scale. In addition, it remains to be clarified whether the scale structure is better represented by the two factors originally proposed (Penza-Clyve & Zeman, 2002) or just by a general factor, as suggested in some studies (Desrosiers et al., 2015; Long et al., 2013; Nitkowski et al., 2019). Therefore, more studies are needed to clarify this question. Moreover, there are no studied measures for the Portuguese population that assess the construct of emotion expression. Such measure is needed to explore the transdiagnostic role of emotional expression in the Portuguese population, and it would allow the clinical assessment of this process that has proved to be transdiagnostically important for child psychopathology.

Therefore, the first goal of the present study is to explore the factor structure of the Portuguese version of the EESC by examining three competing models. First, and according to Penza-Clyve and Zeman (2002), we examined a correlated two-factor model in which Emotion Awareness and Expressive Reluctance would be distinct but correlated factors. Second, and following the results of previous studies, which found a unifactorial structure of the EESC (Desrosiers et al., 2015; Long et al., 2013; Nitkowski et al., 2019), we examined a one-factor model (Difficulties in Emotion Expression). Third, we decided to additionally examine a bifactor model to contribute to clarifying whether the EESC comprises a general factor that explains some proportion of common item variance for all items (Difficulties in Emotion Expression) and/or two specific and orthogonal factors that account for the unique influence of the specific domains over and above the general factor (Emotion Awareness and Emotion Expression factors).

The second goal of this study was to find evidence regarding the validity of the EESC based on its associations with variables that are expected to be related to emotion expression. Based on a previous investigation (e.g., McLaughlin et al., 2011; Scott et al., 2018), the EESC was expected to be positively correlated with internalizing symptoms, namely, anxiety and depression. Moreover, as previous studies found positive correlations between emotion awareness and child rumination (e.g., McLaughlin et al., 2011) and between expressive reluctance and cognitive distortions (Scott et al., 2018), we expect that EESC would be negatively correlated with child mindfulness skills (i.e., the awareness of the present moment, including emotions, and the acceptance of internal states (Kabat-Zinn, 2003). Therefore, based on previous studies which found that the EESC subscales were associated with difficulties in the expression and management of strong emotions (e.g., Christian, 2012) and that expressive reluctance was associated with lower social skill competence (Scott et al., 2018), we also expect the EESC to be positively correlated with behavioral avoidance. Specifically, we expect children who tend to avoid emotional expression and to present difficulties in recognizing emotional states to also avoid situations that may elicit strong emotions.

## Method

### Participants and Procedure

The participants were 286 children (52.1% girls and 47.6% boys) with a mean age of 9.58 years (*SD* = 1.27, range = 8–12), and they were recruited from third (31.8%), fourth (19.9%), fifth (23.1%), and sixth (25.2%) grades of six public schools in central Portugal.

Authorization for the sample collection was obtained from the Ethics Committee of the Faculty of Psychology and Education Sciences of the University of Coimbra and the Board of Directors of Schools. A total of 588 children of six public schools in central Portugal were invited to participate in a study about emotion regulation between December 2019 and March 2020. To be included in the study, children had to be Portuguese, aged between 8 and 12 years, and without a cognitive disability or any learning difficulties whose severity could prevent the correct understanding of the questionnaire’s items (as assessed by student’s teachers). Parents received, through their children’s teachers, the informed consent form and a letter explaining the study objectives and the ethical issues underpinning the study. A total of 306 parents returned informed consent a week later through their children (the remaining parents did not return informed consent or did not allow their children to participate in the study). On the day that the questionnaires were administered to the class eight children missed school. Therefore, 298 children (who have been authorized by their parents and verbally assented their own participation) completed the research protocol in their classroom in 50-min sessions in the presence of a researcher. Questionnaires were read aloud to assist children who presented reading difficulties. Of the 298 questionnaires completed, 12 questionnaires were eliminated due to randomness of responses (e.g., all the answers rated in the same number; zigzag response pattern; visible distraction during the administration; e.g., Krosnick & Presser, 2010), resulting in a total of 286 valid protocols.

### Measures

Children completed a sociodemographic form, developed by the authors, assessing their age, sex, school’s name, and school’s grade.

#### Emotion Expression

The EESC (Penza-Clyve & Zeman, 2002) has 16 items rated on a 5-point Likert-type scale ranging from 1 (*not at all true*) to 5 (*extremely true*). The EESC is composed of two factors consisting of 8 items each: (a) poor awareness, which describes difficulties in labeling internal emotional experiences (e.g., “Sometimes I just do not have the words to describe how I feel”) and (b) expressive reluctance, which describes lack of motivation or willingness to communicate or express negative emotions to others (e.g., “When I’m sad, I try not to show it”). Higher scores indicate more difficulties in emotion expression, namely, poorer emotion awareness and greater reluctance to express emotions.

Authorization from the authors of the original EESC to translate and validate the questionnaire was obtained. Then, two Portuguese researchers independently translated the items of the EESC from English to Portuguese. The two translations were compared, and the similarities and differences of these two versions were discussed, resulting in a first preliminary Portuguese version. Subsequently, the Portuguese version was translated back into English and compared with the original version. As a result, a final comprehensible version that was conceptually consistent with the original version was obtained.

#### Child Anxiety and Depression

The Revised Child Anxiety and Depression Scale—Short Form (Ebesutani et al., 2012) has 25 items rated on a 4-point scale ranging from 0 (*never*) to 3 (*always*) and yields two subscale scores: (a) Depression (10 items; e.g., “I feel sad or empty”) and (b) Anxiety (15 items) distributed across five domains with 3 items each: separating anxiety disorder (e.g., “I am afraid of being in crowded places”), generalized anxiety disorder (e.g., “I worry that something bad will happen to me”), panic disorder (e.g., “suddenly become dizzy or faint when there is no reason for this”), social phobia (e.g., “I worry what other people think of me”), and obsessive-compulsive disorder (e.g., “I have to do some things over and over again, like washing my hands, cleaning or putting things in a certain order”). In the present study, the anxiety domains were not used. Higher scores for anxiety and depression factors indicate higher levels of anxiety and depression, respectively. The reliability of this instrument in the current sample was α = .75 for the depression subscale and α = .86 for the anxiety subscale.

#### Child Mindfulness Skills

The Mindfulness Measure for Children and Adolescents ( Cunha et al., 2013; Greco et al., 2011) contains 10 items rated on a 5-point scale, ranging from 0 (*never*) to 4 (*always*) assessing mindfulness skills in children and adolescents (e.g., “I get upset with myself for having feelings that don’t make sense”). All of the items are reverse scored. Higher scores indicate higher levels of acceptance and mindfulness skills. In the present study, the Cronbach’s α value was .75.

#### Child Behavioral Avoidance

The Child Avoidance Measure–Self Report (Whiteside et al., 2013) is a single-factor self-report measure composed of 8 items to assess children’s tendency to avoid stimuli that elicit anxiety, fear, or worry. The questionnaire presented a stem statement (“When I feel scared or worried about something. . .”), and children responded to items using a 4-point Likert-type scale ranging from 0 (*almost never*) to 3 (*almost always*) to indicate how well each item describes the way that he or she usually reacts when he or she feels “scared or worried about something.” Items include passive avoidance, active refusal, delay, and expressing anger (e.g., “I try not to go near it”; “I refuse to do it”). Higher scores indicate higher levels of behavioral avoidance. In the present study, the Cronbach’s α value was .85.

### Data Analysis

Data and statistical analyses were performed using the IBM Statistical Package for the Social Sciences (SPSS) for Windows (version 26.0, Armonk, NY: IBM Corp.) and AMOS (version 24 Chicago: IBM SPSS). There were no missing values. Descriptive statistics were calculated to explore the sample’s sociodemographic characteristics.

#### Confirmatory Factor Analysis

**Figure 1.**
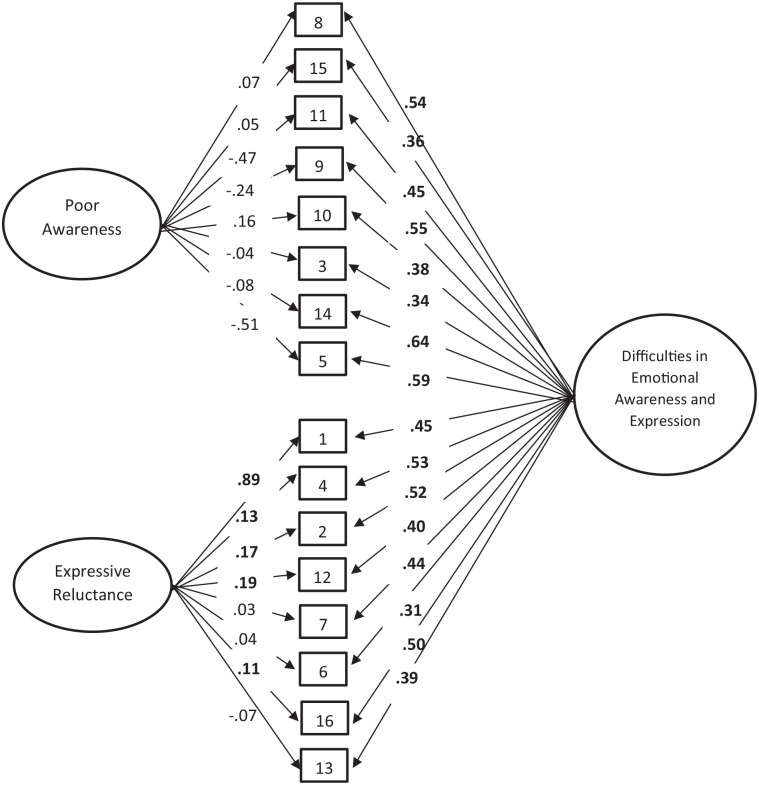
Standardized Factor Loadings for the Bifactor Confirmatory Model With Two Specific Factors *Note.* Factor loadings in bold were significant (all of them had *p* values < .001, except factor loadings for items 4, 2, and 16 in the Expressive Reluctance Factor, which presented *p* values <.005). The remaining items (not in bold) did not load significantly on the factor (i.e., all the items of poor awareness factor and items 7, 6 and 13 of expressive reluctance factor).

A CFA using maximum likelihood estimation was conducted to test the adequacy of the factor structure of the EESC to the Portuguese population. Three models were estimated: (a) a correlated two-factor model corresponding to the two factors found in the original EESC (Poor Awareness and Expressive Reluctance); (b) a one-factor model in which all items loaded on a single factor (Difficulties in Emotion Expression); and (c) a bifactor model (see Figure 1). In the bifactor model, all items loaded on a general factor (Difficulties in Emotion Expression) with nonzero loadings on the domain-specific factor that they were designed to measure and zero loadings on the other factors. In addition, the two specific factors (Emotion Awareness and Expressive Reluctance) were not correlated with each other, and error terms that were associated with each item were not correlated.

The model fit was assessed through the comparative fit index (CFI), the root-mean-square error of approximation (RMSEA), and the standardized root-mean-square residual (SRMR). The fit of the models was evaluated based on a two-index approach: combination of an SRMR of .08 or lower with either the RMSEA of .06 or lower or with the CFI of .95 or higher for an adequate fit (Hu & Bentler, 1999). To compare the models, the difference of χ^2^ (Δχ^2^) and the Akaike information criterion (AIC; Akaike, 1987) was used. A significant Δχ^2^ indicates that the model with the lowest χ^2^ presents a better fit and the model with the smallest AIC values was considered the best-fitting model (Kline, 2011).

Factor loadings for the three tested models were analyzed. Factor loadings of .32 or above were considered meaningful (Tabachnick et al., 2007). If factor loadings of the general factor in the bifactor model are similar to the factor loadings of the one-factor model and the factor loadings of the specific dimensions in the bifactor model are lower than the correlated two-factor model, it suggests a high influence of the general factor on the items variance and a minor influence of the specific dimensions (Zwaanswijk et al., 2017).

#### Bifactor Model-Based Psychometric Indices

Several bifactor model-based psychometric indices were computed: the explained common variance (ECV; Sijtsma, 2009; Ten Berge & Sočan, 2004), the percentage of uncontaminated correlations (PUC; Bonifay et al., 2015; Reise et al., 2013b), and the omega reliability coefficients, including the omega (ω), omega subscale (ωS), omega hierarchical (ωH), omega hierarchical subscale (ωHS), and relative omega (ωH/ω; McDonald, 1999; Reise, 2012; Zinbarg et al., 2005)

The ECV is an index of the degree of unidimensionality and assesses the relative strength of the general factor or the proportion of all common variance explained by the general factor (Rodriguez et al., 2016a, 2016b). Higher values of ECV indicate little common variance beyond the variance accounted for by the general factor, suggesting a strong general factor and, thus, unidimensionality (Reise et al., 2013a). The ECV is calculated for general factor and specific factors (only relative to items loading on that factor). According to Rodriguez et al. (2016a), “ECV values closer to 1.0 indicate a strong general factor and that the common variance is essentially unidimensional” (p. 231). The PUC is another strength index, and higher values suggest that the parameter estimates in a unidimensional model are less likely to be biased (Reise, 2012; Rodriguez et al., 2016b). According to Rodriguez et al. (2016a) “when ECV is >.70 and PUC >.70 relative bias will be slight, and the common variance can be regarded as essentially unidimensional” (p. 232).

Omega coefficients, namely, ω, ω*H*, and ωH/ω were calculated to evaluate reliability. ω is a factor-analytic model-based index of internal reliability. It is the ratio of a measure’s estimated true score variance (i.e., variance due to factors) to the measure’s estimated total score variance (i.e., variance due to the factors and their uniqueness; Reise et al., 2013a; Rodriguez et al., 2016b). For the general factor, all items were considered (ω); for specific factors, only items loading on that factor were considered (ωS). ωH and ωHS compare the variance of only one construct (general factor or domain-specific factor) to the total score variance. Therefore, while ω provides an estimate of the amount of the score variance due to all common factors, ωH and ωHS estimate the amount of the score variance due to a single common factor (i.e., the general or specific factor; Reise et al., 2013b). The ωHS reflects the proportion of reliable systematic variance of a given subscale score after partitioning out variability attributed to the general factor (Reise et al., 2013a; Rodriguez et al., 2016a). We also computed the relative omega (ωH/ω), which is the ωH divided by omega.

Higher values of ωH indicate that the general factor is the dominant source of systematic variance. An ωH >.50 and closer to .75 is suggestive of factor strength (Hancock & Mueller, 2001; Reise et al., 2013a). According to Rodriguez et al. (2016a), an ωH of .80 or above indicates that total scores can be considered essentially unidimensional. The relative omega (ωH/ω) corresponds to the percentage of the reliable variance in the multidimensional composite due to the general factor and the percentage of reliable variance in the subscales composite that is independent of the general factor.

According to Reise et al. (2013), when PUC values are <.80 but general ECV values are >.60 and the ωH for the total score is >.70, the presence of multidimensionality is not considered severe enough to disqualify the interpretation of the measure as essentially unidimensional.

#### Validity of the EESC

The validity of the EESC scores was explored based on their relation with variables expected to be associated with EESC (child’s levels of anxiety and depression, mindfulness skills, and child behavioral avoidance). Correlations around .10 were considered small; correlations near .30 were considered medium; and correlations of .50 or higher were considered large (Cohen, 1988).

## Results

### Confirmatory Factor Analyses

The results of the model fit of the three competing models are presented in Table 1. The three tested models presented an acceptable fit to the data. However, the bifactor model presented a significantly better fit to the data than the one-factor model, Δχ^2^ (15) = 70.42 (*p* < .001), and the correlated two-factor model, Δχ^2^ (15) = 70.42 (*p* < .001). Congruently, the bifactor model presented lower values for AIC than the other two models, indicating a better model fit. Also, the other fit statistics are better for the bifactor model (i.e., lower values of RMSEA and SRMR; higher values of CFI). The one-factor model and the correlated two-factor model presented similar values of model fit (i.e., same value of RMSEA and SRMR; similar value of CFI); however, the correlated two-factor model presented a significantly better fit to the data the one-factor model, Δχ^2^ (1) = 7.56 (*p* = .006). Significant and high latent factor intercorrelations were found in the correlated two-factor model (*r =* .88).

**Table 1:** Fit Statistics for the Confirmatory Factor Analysis.

Model	χ^2^(*df*)	*p*	CFI	SRMR	RMSEA [95% CI]	AIC
Correlated two-factor	214.57 (103)	< .001	.87	.06	.06 [.049, .073]	277.99
One-factor model	222.13 (104)	< .001	.86	.06	.06 [.052; .075]	286.13
Bifactor model	151.71 (89)	< .001	.92	.05	.05 [.036, .063]	245.71

*Note.* CFI = comparative fit index; SRMR = standardized root-mean-square residual; RMSEA = root-mean-square error of approximation; AIC = Akaike information criterion; CI = confidence interval.

### Factor Loadings and Dimensionality

All standardized factor loadings of the correlated two-factor model and one-factor model were significant (*p* < .001). Factor loadings of the correlated two-factor model ranged from .32 to .67. Factor loadings of the one-factor model ranged from .31 to .66.

Standardized factor loadings of the bifactor model are presented in Figure 1. All items loaded significantly on the general factor (*p* < .001), and factor loadings ranged from .31 to .64. These values were similar to those in the one-factor model. In contrast, factor loadings of the subscales in the bifactor model were meaningfully lower than in the correlated two-factor model. Indeed, none of the items from the Poor Awareness subscale loaded significantly on that specific factor and the majority of them were meaningful (factor loadings ranging from −.04 to – 08, except for Item 5 and 11 with factor loadings of .51 and .47, respectively). Similarly, all the items’ factor loadings from the Expressive Reluctance subscale, except for item 1 (“*I prefer to keep my feelings to myself”*), were not meaningful with factor loadings ranging from .03 to .19. Moreover, the loadings of Items 6 *(“I usually do not talk to people until they talk to me first”)*, 7 *(“When I get upset, I am afraid to show it”*), and 13 (*“Other people don’t like it when you show how you really feel”*) were nonsignificant. Therefore, in the bifactor model, almost all items loaded more strongly on the general factor than on each specific factor. The only exception was Item 1, which loaded more strongly on the specific factor than on the general factor. This pattern of results suggests that most of the items’ variance is shared with the general factor, with the exception of Item 1.

As presented in Table 2, the ECV for the general factor was .707 (PUC = .533), which suggests that the general factor explains a high proportion of the common variance extracted (above 70%). The ECV of the Expressive Reluctance factor was .357, and that of the Poor Awareness factor was .229, which suggests that these factors explain a lower proportion of the respective items’ common variance (about 36% and 23%, respectively).

**Table 2.** Indices of the Bifactor Model.

Bifactor model	ECV	ω; ωS	ωH; ωHS	ωH/ω; ωHS/ωS
Total score	.707	.841	.793	.943
Poor Awareness	.229	.754	.052	.070
Expressive Reluctance	.357	.729	.109	.149

*Note.* ω, ωH, and ωH/ω are indices for the total score; ωS, ωHS, and ωHS/ω are omega indices for the subscales. ECV = explained common variance. ω = omega; ωS = omega subscale; ωH = omega hierarchical; ωHS = omega hierarchical subscale; ωH/ω = relative omega.

### Reliability Indices for the Bifactor Model

As presented in Table 2, ω values were above .80 for the general factor and above .70 for the two subscales indicating a good and acceptable reliability, respectively. The ωH index was .793 for the total score, .052 for the Poor Awareness factor, and .109 for the Expressive Reluctance factor, which is suggestive of factor strength. In the same way, the relative omega indicates that 94% of the reliable variance is due to the general factor (ωH/ ω = .943) and only 7% of the reliable variance of the Poor Awareness factor (ωH/ ω = .070) and 14.9% of the reliable variance of the Expressive Reluctance factor (ωH/ ω = .149) are independent of the general factor. These results suggest a strong general factor and support the computation of a Difficulties in Emotion Expression total score. The amount of reliable systematic variance of the subscale scores of Poor Awareness and Expressive Reluctance after partitioning out variability attributed to the general factor was low, which does not support the use of these factors as independent subscales.

### Item Descriptives

The means and standard deviations of each item and its correlations with the scale total score of the EESC are presented in Table 3. Almost all items presented medium to strong correlations with the scale total score.

**Table 3.** Item and Scale Descriptives and Item-Total Correlations.

Item no.	*M* (*SD*)	Item-total correlation
Item 1	3.18 (1.20)	.46
Item 2	2.85 (1.24)	.48
Item 3	2.78 (1.40)	.30
Item 4	3.18 (1.40)	.50
Item 5	3.12 (1.47)	.55
Item 6	2.33 (1.42)	.29
Item 7	2.20 (1.23)	.38
Item 8	2.73 (1.43)	.48
Item 9	3.00 (1.40)	.51
Item 10	2.22 (1.40)	.32
Item 11	3.08 (1.33)	.43
Item 12	2.20 (1.23)	.39
Item 13	2.03 (1.22)	.34
Item 14	2.86 (1.44)	.58
Item 15	2.59 (1.47)	.31

### Validity of the EESC in Relation to Other Variables

The correlations between the EESC total score (Difficulties in Emotion Expression) and other variables were analyzed to explore the construct validity of the scale. As presented in Table 4, all correlations were statistically significant and were considered medium to large, except the correlations of the EESC with behavioral avoidance, which were considered small (although significant).

**Table 4.** Correlations Between the EESC and Child Depression, Anxiety, Mindfulness Skills, and Behavioral Avoidance.

Variable	Difficulties in emotional expression (EESC)
Child Anxiety	.53**
Child Depression	.46**
Child Mindfulness skills	−.51**
Child Behavioral Avoidance	.17**

*Note.* EESC = emotion expression scale for children.

** *p* < .01.

## Discussion

The EESC is a 16-item self-report questionnaire for children developed by Penza-Clyve and Zeman (2002) to assess children’s difficulties in emotion expression, namely, difficulties in being aware of one’s emotions and unwillingness to express emotions. Although this scale has been widely used in many studies (e.g., Allen et al., 2012; Trosper & Ehrenreich May, 2011), it has only been validated among American (Penza-Clyve & Zeman, 2002) and German adolescents (Nitkowski et al., 2019). Moreover, the results regarding the EESC factorial structure are not congruent, and thus, it was still unclear whether the scale is better represented by two factors (Penza-Clyve & Zeman, 2002) or by a general factor (e.g., Nitkowski et al., 2019). Therefore, there was no consensus about the use of its total score or the two subscales’ scores in subsequent studies. Moreover, there are no studied measures for the Portuguese population to assess emotion expression, which compromises the research and clinical practice in this area. To respond to these gaps, the present study aimed to examine the factor structure of the Portuguese version of the EESC. Three models were analyzed: (a) a correlated two-factor model composed of Poor Emotion Awareness and Expressive Reluctance subscales (as proposed by Penza-Clyve & Zeman, 2002); (b) a one-factor model composed by a single factor (Difficulties in Emotion Expression), as proposed by Nitkowski et al. (2019) and also suggested in other studies (Desrosiers et al., 2015; Long et al., 2013); and (c) a bifactor model examined whether EESC would be better explained by a general factor of “Difficulties in Emotion Regulation” and/or by two specific and orthogonal factors corresponding to the two subscales. In addition, the validity evidence of the EESC was examined based on its associations with other variables of children’s socioemotional functioning.

With regard to the factor structure of the scale, although all the analyzed models presented an adequate fit to the data, the bifactor model provided a significantly and noticeably better fit than the competing models, supporting the bifactor structure of the EESC.

The results of the bifactor model support a general factor of “difficulties in emotion expression” that is reliably measured by the EESC total score and that separately calculating the subscale scores of the EESC is questionable. This is supported by the results discussed below.

First, high intercorrelations were found among the two latent variables in the correlated two-factor model (and thus, redundant and correlated with the total score), and all the EESC items were moderately or highly associated with the total score which highlights the interrelatedness of these dimensions, reinforcing the use of the EESC total score.

Second, in the bifactor model, all items loaded significantly on the general factor, and all items (except Item 1) loaded more strongly on the general factor than on the respective specific factor. Moreover, the factor loadings of the general factor in the bifactor model were similar to the factor loadings of the one-factor model, while the factor loadings of the dimensions in the bifactor model were lower than the factor loadings of the correlated model. According to Zwaanswijk et al. (2017), this pattern of results suggests that the general factor has a greater influence on the items’ variance while the two dimensions have a minimal influence.

Third, the unidimensionality strength indices of the bifactor model (e.g., ωH = .79; ECV = .71; PUC = .53 for the total score) indicate that the presence of multidimensionality is not severe enough to disqualify the interpretation of the EESC as essentially unidimensional (Reise et al., 2013b) and that the general factor explains approximately 71% of the common variance. Indeed, ω indicates better reliability for the general factor than for the dimensions. Moreover, although the reliability for the subscales is acceptable, after controlling for the variance associated with the general factor, the two dimensions explained little variance beyond that explained by the general factor (ωHS). Indeed, values of ωHS (Poor Awareness = .052; Expressive Reluctance = .109) were below the threshold of .50 recommended by Reise et al. (2013a) to consider a subscale a valid representation of a separable dimension. If we compare the subscales’ indices, the Expressive Reluctance subscale presented a higher ω*H* (.149) and ECV (.36) than the Poor Awareness subscale (ω*H*= .09; ECV = .23), which indicates that the Expressive Reluctance subscale captures a more substantial proportion of specific variance. In contrast, the indices of the Poor Awareness subscale suggest that this dimension almost overlaps with the general factor of Emotion Expression. Moreover, the results indicate that 94.3% of the common variance is explained by a general factor of “Difficulties in Emotion Expression” (ωH/ ω = .943), while only 7% of the reliable variance of the Poor Awareness subscale (ωH/ ω = .070) and 14.9% of the reliable variance of the Expressive Reluctance subscale (ωH/ ω = .149) seem to be independent of the general factor.

These results globally suggest the presence of a strong general factor of “Difficulties in Emotion expression” and, consequently, the computation of a scale’s total score. Although the dimensions do explain some variance over and above the general factor, “it is arguable that the subscales scores provide no added value beyond the total score” (p. 131), and thus there would be no support for reporting separate subscales scores (Reise et al., 2013a).

Indeed, the scale seems to be more reliable for evaluating the overall construct of Emotion Expression (even if it is assumed that there is multidimensionality—with a small percentage of the variance to be explained by the subscales). These results are supported by the theoretical background in which emotion expression, as a broader concept, is composed not only of emotional responses (such as the reluctance to express emotions) but also of the recognition and awareness of internal experiences—emotion awareness (Gross & Thompson, 2007; Penza-Clyve & Zeman, 2002).

Likewise, if a researcher aims to assess the motivation or reluctance to express emotions, we recommend the use of the total score of the EESC (instead of the use of the Reluctance to Express Emotion subscale) as the willingness to express/repress emotions is influenced by emotion awareness and involves it as a prior stage. Namely, emotion awareness (which includes an attitudinal aspect responsible for evaluating emotions—beliefs about emotions) is a prerequisite for the motivation to express emotions; thus, a child may express or repress their emotions according to his or her beliefs about whether emotions should be expressed or repressed (e.g., Rieffe & De Rooij, 2012; Rieffe et al., 2008). Moreover, to be able to express emotions properly, one has to be first able to recognize, identify, and evaluate the emotions (e.g., Gross, 1999). Thus, when a researcher aims to evaluate the child’s motivation to express emotions, this evaluation should include the assessment of the child’s emotion awareness, which is consistent with the results of the present study on the unidimensionality of the EESC.

On the contrary, a researcher may aim to evaluate the construct of emotion awareness independently, as it is an intrinsic process and a prior stage to emotion expression; thus, it could not necessarily involve an outward display (Croyle & Waltz, 2002; Lane et al., 1998). A child could be able to identify and be aware of his or her emotions but still not be motivated to express them. However, in this case, if a researcher aims to evaluate only this first stage of the emotion expression process, we consider that it would be more adequate to use another scale, such as the Emotion Awareness Questionnaire (Rieffe et al., 2008) or the Levels of Emotion Awareness Scale (Lane et al., 1998)), as the results of the present study indicate that the EESC seem to be more reliable to access the general factor of Emotion Expression and that the Poor Awareness subscale seems to almost overlaps with the general factor.

Although the scale was originally named the “Emotion Expression Scale” (Penza-Clyve & Zeman, 2002), the general factor found in the present study was named “Difficulties in Emotion Expression” as items of the EESC are presented in a negative way and are not reversed (e.g., “I do not like to talk about how I feel”); thus, we believe that this name would better convey the real content and purpose of the scale. Although the designation, “Low Emotion Awareness/Suppression,” adopted by Nitkowski et al. (2019), also conveys the objective and content of the scale, we chose to remain as faithful as possible to the name of the original scale.

Finally, the statement that the EESC is fundamentally unidimensional and that it measures a general factor of Emotion Expression needs further research and clarification. Indeed, this is the first study analyzing the bifactor structure of the EESC and some of the results suggest the unidimensionality but are not undoubted (e.g., ECV values of .71 and PUC of .53, in combination with the omega coefficients, suggest the unidimensionality, but, to assume that the common variance is essentially unidimensional both should be above .70) and the subscales still explain some of the variance (especially Expressive Reluctance subscale). Thus, further research is needed to determine the (uni) dimensionality of the EESC.

### The Association of the EESC With Other Variables of Children’s Socioemotional Functioning

The second main goal of the present study was to examine the validity of the EESC by analyzing the association with other variables that were expected to be correlated with emotion expression. All the correlations were statistically significant and in the expected direction. Specific relations are discussed below.

First, as expected, higher levels of difficulties in emotion expression were significantly and largely associated with higher levels of internalizing symptoms, namely, anxiety and depression. This result is congruent with previous studies in which emotion awareness and expressive reluctance (assessed by EESC) were also significantly correlated with measures of depressive and anxiety symptoms (Brockenberry, 2016; Kranzler et al., 2016; McLaughlin et al., 2011). In addition, other studies using other measures to assess emotion awareness (e.g., Berthoz et al., 2000; Southam-Gerow & Kendall, 2000; Zeman et al., 2002) and studies using other measures to assess reluctance to express emotions (e.g., Betts et al., 2009; Feng et al., 2009; Larsen et al., 2013; Suveg & Zeman, 2004; Zeman et al., 2001) also found an association of these variables with internalizing symptoms, namely, with childhood anxiety disorders and depression. Therefore, the evidence seems to consistently suggest that difficulties in emotion expression are associated with greater anxious and depressive symptomatology. Thus, the promotion and development of children’s ability to identify, recognize, tolerate, and adequately express their emotions may be a protective factor against psychopathology.

Second, as expected, EESC was also significantly, negatively, and largely associated with child mindfulness and acceptance skills. Mindfulness involves having awareness of the present moment, including the internal and external world of the child. Therefore, it involves the awareness of emotions and an accepting and nonjudgmental attitude toward emotional states rather than emotional avoidance. Consequently, it could be expected that children with higher levels of mindfulness skills would have fewer difficulties in emotion expression (i.e., more ability to be aware of their emotions and to express them instead of repressing/avoiding them). Previous studies also found positive correlations between EESCs and child rumination (e.g., McLaughlin et al., 2011) and cognitive distortions (Scott et al., 2018), which are variables that are expected to be negatively associated with mindfulness (Sears & Kraus, 2009; Svendsen et al., 2017). These results could suggest that the promotion of children’s mindfulness skills (e.g., through a mindfulness-based intervention) may be associated with the promotion of higher levels of emotion awareness and provide tools that allow adequate emotion expression.

Third, higher levels of difficulties in emotion expression were also significantly associated with higher levels of behavioral avoidance. Previous research found that poor emotion awareness in children is related to difficulties in regulating and managing negative emotions (Christian, 2012; Penza-Clyve & Zeman, 2002; Saarni, 1999), and consequently, they may avoid situations that they think may elicit strong emotions because they probably do not know how to identify or manage their feelings in such situations. In contrast, if children are able to recognize and adequately express their emotional states, they are likely more able to manage them more easily and thus adopt more coping behaviors in challenging situations. However, although this association was significant and occurred in the expected direction, its magnitude was small (in contrast to all the other associations studied that were large). This may indicate that EESC could be more associated with internalizing variables (e.g., anxiety, depression, mindfulness) than with more externalizing variables, such as behavior avoidance. In addition, this result can also be justified by the fact that some children who have more difficulties in emotion expression may try to deal with situations that provoke strong emotions (instead of avoiding it) to avoid showing their emotions and vulnerability to others, even if internally they are very emotionally activated. This would happen in children who adopt more compensatory processes in relation to their emotions (Young & Lindemann, 1992).

### Contributions and Limitations

The present study has important methodological, clinical, and theoretical contributions. First, it contributes to addressing the gap related not only to the few existing studies assessing EESC psychometric properties but also to the inconsistency related to the EESC factorial structure and dimensionality (which leads to no agreement about the use of the total score of the EESC or the use of the two subscales in the following studies and clinical practice). The present study is the second aimed at analyzing the EESC’s factorial structure and its psychometric properties and the first analyzing a bifactor model of the EESC which is a type of analysis more robust and informative than simply comparing a one-dimensional model with a correlated model. The results of this study indicate that the factorial structure of the EESC is better explained by a bifactor model and suggest a strong general factor of “Difficulties in Emotion Expression,” thus supporting the use of the total score of the EESC instead of the use of the two subscale scores. However, as already mentioned, further research using bifactor analysis is needed to determine the (uni)dimensionality of the EESC.

Second, this study offers to the Portuguese scientific community a reliable measure to assess children’s difficulties in emotion expression. The EESC is a short measure that is easy to apply and evaluates a construct that seems to be quite involved in child psychopathology and mental health (as suggested by the results of the associations with other variables in the present study and the results of previous studies using the EESC). Therefore, the EESC is a relevant measure for further studies and the clinical context, allowing the understanding of the transdiagnostic processes behind children’s psychopathology and mental health.

Third, these results also have theoretical implications, corroborating the hypothesis that emotion expression is composed not only of the motivation to express or repress emotions but also by emotion awareness.

Finally, the results of the present study also have additional clinical contributions as they highlight that children who have difficulties in emotion expression seem to present higher levels of internalizing symptomatology and behavioral avoidance, which may mean that emotion expression may be a transdiagnostic mechanism relevant for the prevention of psychopathology and the promotion of child involvement in coping behaviors. Moreover, the results of the present study also seem to indicate that children with higher levels of difficulties in emotion expression present lower mindfulness skills, which may indicate that mindfulness training (e.g., Black et al., 2009) may be useful to promote emotion awareness and emotional expression in children. Other emotion-focused intervention programs that combine mindfulness strategies with other cognitive–behavioral strategies may be useful to prevent psychopathology and promote mental health in children as they are focused on the promotion of emotion awareness and adequate emotion expression (e.g., Unified Protocol for Transdiagnostic Treatment of Emotional Disorders in Children; Ehrenreich-May et al., 2017).

Despite the important contributions of the present study, it also has some limitations that should be acknowledged. First, the EESC was only administered once; thus, we were not able to determine the test–retest reliability. Second, the sample included only children in schools in central Portugal, which limits the generalization of results to children in other areas of the country. Moreover, these results may be limited to Portuguese culture. Portuguese population is a Latino culture that is usually characterized for being warm and emotive. Thus, the motivation and reluctance to express emotions may differ from other cultures, especially from non-Latino cultures. Indeed, previous studies found that culture and context influence children’s socioemotional development (Cole et al., 1994; Eisenberg et al., 1998a, 1998b). Thus, the bifactor model should be tested also in other cultures and culture differences explored. Third, although this measure proved to be robust for evaluation in the general population, it is considered essential to assess its suitability for the clinical population. Fourth, it would be interesting and relevant to assess the associations between the EESC and measures of externalizing psychopathology, in addition to the measures assessing internalizing symptoms used in this study. Finally, although the hypotheses formulated based on the correlations between EESC and other measures represent a starting point for future research, it is important that future studies adopt a longitudinal design and analyze the mediating and moderating role of these variables on psychopathology and mental health.
